# Metabolism Disrupting Chemicals and Alteration of Neuroendocrine Circuits Controlling Food Intake and Energy Metabolism

**DOI:** 10.3389/fendo.2018.00766

**Published:** 2019-01-09

**Authors:** Marilena Marraudino, Brigitta Bonaldo, Alice Farinetti, GianCarlo Panzica, Giovanna Ponti, Stefano Gotti

**Affiliations:** ^1^Neuroscience Institute Cavalieri Ottolenghi, Turin, Italy; ^2^Department of Neuroscience “Rita Levi-Montalcini”, University of Turin, Turin, Italy; ^3^Department of Veterinary Sciences, University of Turin, Turin, Italy

**Keywords:** metabolic disruptor, food intake, hypothalamus, estrogens, bisphenol A, tributyltin, genistein

## Abstract

The metabolism-disrupting chemicals (MDCs) are molecules (largely belonging to the category of endocrine disrupting chemicals, EDCs) that can cause important diseases as the metabolic syndrome, obesity, Type 2 Diabetes Mellitus or fatty liver. MDCs act on fat tissue and liver, may regulate gut functions (influencing absorption), but they may also alter the hypothalamic peptidergic circuits that control food intake and energy metabolism. These circuits are normally regulated by several factors, including estrogens, therefore those EDCs that are able to bind estrogen receptors may promote metabolic changes through their action on the same hypothalamic circuits. Here, we discuss data showing how the exposure to some MDCs can alter the expression of neuropeptides within the hypothalamic circuits involved in food intake and energy metabolism. In particular, in this review we have described the effects at hypothalamic level of three known EDCs: Genistein, an isoflavone (phytoestrogen) abundant in soy-based food (a possible new not-synthetic MDC), Bisphenol A (compound involved in the manufacturing of many consumer plastic products), and Tributyltin chloride (one of the most dangerous and toxic endocrine disruptor, used in antifouling paint for boats).

## The Hypothalamic Control of Food-Intake and Energy Metabolism

**Figure 1:**
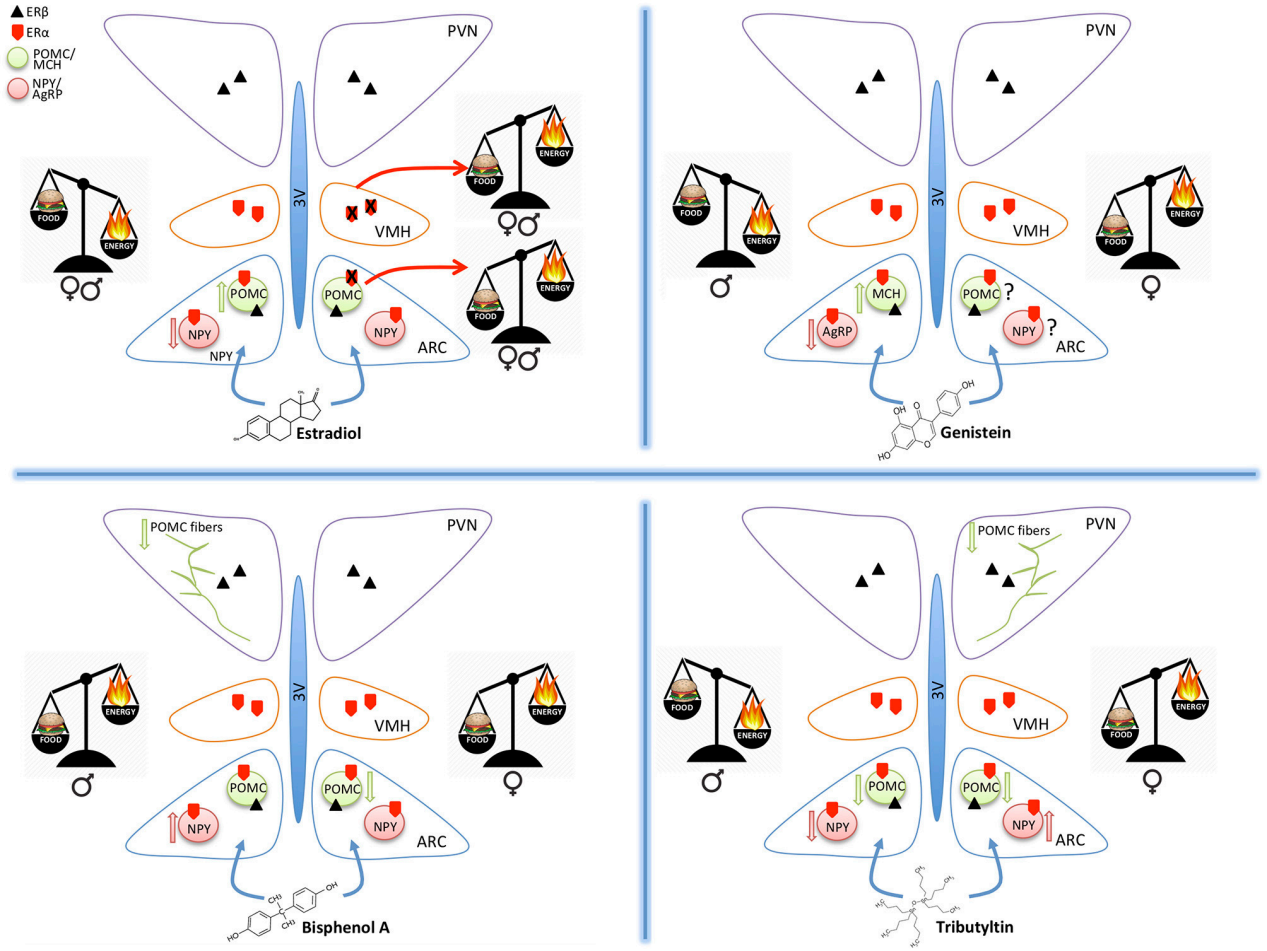
Schematic drawings summarizing the effect of estradiol, genistein, bisphenol A, and tributyltin on the orexigenic (NPY/AgRP) and anorexigenic (POMC/MCH) neurons of the hypothalamic arcuate nucleus (ARC), projecting to the ventromedial (VMH) and paraventricular (PVN) nuclei. All these nuclei contain estrogen receptors α and β mediating the effects of estradiol and of the other substances. The exposure to different molecules induces an imbalance in food intake (food) or energy expenditure (energy). These effects may be similar in both sexes (estradiol), or different (the other MDC) and are mediated by effects on the two main populations of ARC (up arrow = increased expression, down arrows = decreased expression). POMC projections to PVN are also affected.

The hypothalamus plays an essential role in controlling food intake and energetic status, mainly through two antagonistic neuronal populations of the hypothalamic arcuate nucleus (ARC): the orexigenic neurons (appetite-stimulating), characterized by the co-expression of agouti-related peptide (AgRP) and neuropeptide Y (NPY), and the anorexigenic neurons (appetite-suppressing) that co-express pro-opiomelanocortin (POMC) and cocaine- and amphetamine-regulated transcript (CART) (1–4) (Figure 1). These ARC neurons project to other hypothalamic nuclei, among which the Ventromedial hypothalamic (VMH), and the Paraventricular (PVN) nuclei (Figure 1). The latter one is the most important center of metabolic control: it integrates orexigenic and anorexigenic inputs from ARC and modulates energy expenditure through the hypothalamic pituitary adrenal (HPA)-axis (5), and the hypothalamic pituitary thyroid (HPT)-axis (6, 7).

These systems are sensitive to peripheral signals of energetic balance (for example leptin, insulin, and GHrelin). Leptin blood levels depend on the size of fat stores (8) and acts as an anorexigenic factor to adjust energy requirements, fat reserves, and food intake (9). In contrast, GHrelin has an orexigenic role in the central control of appetite and metabolism (10). Moreover, also sexual hormones, thyroid hormones, and growth factors can modulate the hypothalamic circuits regulating appetite, satiety, and metabolism (11). In particular, in mammals, estradiol (E_2_) has an important role on the regulation of food intake and metabolism with an appetite-suppressing effect (12, 13).

Several synthetic or natural molecules that are present in the environment may interact with the estrogen or androgen signaling chain (xenoestrogens, xenoandrogens) and have been classified as endocrine disrupting chemicals [EDCs, (14)]. In addition, many of these EDCs have been considered to belong to the category of metabolism disrupting chemicals (see below), however, little attention has been dedicated, until now, to their action of neural circuits controlling food intake and energy metabolism.

## Central Action of Metabolism-disrupting Chemicals (MDCs)

The metabolism-disrupting chemicals (MDCs) have been defined (Parma Consensus Statement, (15) as those endocrine disrupting chemicals (EDCs) that are able to promote metabolic changes that can result in obesity, Type 2 Diabetes Mellitus (T2DM) or fatty liver in animals including humans. The major targets for these compounds are the fat tissue and the liver (11), however, they may regulate nutrient ingestion and metabolism by altering intestinal transport, secretion of gut peptides, composition of the gut microbiota as well as the expression of hypothalamic neuropeptides that control food intake (11, 16). Several studies reported that MDCs can alter food intake, with different effects based on dose, timing, and exposure duration (17–19). In particular, exposure to MDCs during the perinatal period and/or adulthood modifies the cues that regulate energy homeostasis, such as serum levels of insulin, leptin, and fatty acids (20).

In this short review, we will describe the neuroendocrinological effects of a possible new, not-synthetic, MDC (Genistein), and of two synthetic identified MDCs (Bisphenol A and Tributyltin), (Table 1).

**Table 1:** Summary of the effects of genistein (GEN), bisphenol A (BPA), and tributyltin (TBT) on circuits controlling food intake and energetic status in rodent hypothalamus.

**Compound**	**Experimental model**	**Administration**	**Dose**	**End point**	**Effects SNC**	**Peripheral effects**	**References**
GEN	Male CD1 mice	Orally for 6 weeks to both parents before mating	Soy based food (~190 ppm GEN)	Adult males (3 and 6 months old)	↓ AgRP (mRNA) ↑ MCH (mRNA) ↑ Orexin-A (mRNA) ↑ TRH (mRNA) = NPY (mRNA) = POMC (mRNA) = CART (mRNA)	↓ body weight ↓ adiposity ↓ resistance to cold ↑ lipid oxydation ↑ locomotor activity ↑ muscle mass in males ↑ food intake in males	(21)
BPA	CD-1 mice	Perinatal (GD0 until weaning, PND21)	1 or 20 μg/kg diet	Adult males and females	**Females:** ↓ POMC mRNA in ARC (when combined with HFD) ↑ ERα+ POMC+ cells in ARC ↑ ERα in ARC **Males:** ↓ POMC fiber in PVN ↑ ERα+ POMC cells in ARC ↑ NPY and AgRP expression in ARC (when combined with HFD)	**Dams**: ≠ BW ≠ Food intake **Females:** ↑ in food intake ↑ weight adiposity ↑ leptin plasma level **Males:** ↑ energy expenditure ↑ leptin plasma levelImpaired glucose tolerance	(18)
BPA	CD-1 mice	Perinatal (GD0 until weaning P21)(+GD12 BrdU)	20 μg/kg (0.02 ppm) diet	Males and females pups (PND2-8-10-16-21) + adult (PND130) for leptin measurements	↓ Density of POMC fiber in PVN	Leptin resistanceDelayed postnatal leptin surges	(22)
BPA	Sprague-Dawley rats	Maternal and gestational exposure (2 weeks prior mating and throughout pregnancy)	5 mg/L in drinking water	Males pups at PND1 sacrificed to obtain NPCs+ control NPCs treated *in vitro*.	***In vivo***: ↑ hypothalamic NPCs proliferation and differentiation ↑ neuroproliferative (Hes1) and proneurogenic (Ngn3) protein expression ↑ AgRP/NPY expression ↓ POMC expression***In vitro***: ↑ AgRP/NPY expression ↓ POMC expression ↑neuron/glia ratio ↑ LSD1		(23)
BPS	Swiss Albino mice	From PND21 for 10 weeks of treatment	0-25-50-100 μg/kg/day in drinking water	Adult males	↑AgRP mRNA ≠ POMC, CART and NPY mRNA ↓ APJ mRNA ≠ Apelin mRNA	↑ BW ↑ Food intake ↑ Feed efficiency	(24)
TBT	C57Bl/6	Acute in adult mice	10 mg/Kg of body weight	Adult males	↑*c-fos* expression in ARC		(25)
TBT	C57Bl/6	Chronic in adult mice (from PND90 to PND120)	0.025 mg/Kg of body weight	Adult males and females	**Females:** ↑ NPY in VMH ↓ Y1 transgene expression in PVN and VMH **Males:** ↓ NPY in ARC, PVN and DMH ↓ Y1 transgene expression in ARC, PVN, DMH and VMH (p-value close to significance)	**Females:** ↓ circulating leptin level ↑ feed efficiency **Males:** ↓ circulating leptin level ↑ feed efficiency	(19)
TBT	CD-1 mice	Chronic in adult mice (from PND30 to PND65)	0.025 mg/Kg of body weight	Adult males and females	**Females:** ↓ POMC in ARC, DMH and PVN **Males:** ↓ POMC in PVN		(26)
TBT	Sprague-Dawley rats	Chronic in rats for 54 days	0.5 μg/Kg of body weight	Adult males and females	**Females:** ↑ NPY *m*RNA expression **Males:** ↓ POMC, CART and AgRP *m*RNA expression	**Females:** ↑ food intake	(27)

### Genistein

Soy isoflavones, in particular Genistein (GEN), are very abundant in soy-based food (28) and are an important source of EDCs (29). GEN action requires both estrogen receptor (ER)α and ERβ (30), although, compared to E_2_, GEN affinity is low for ERα, while it is similar for ERβ (31–33). ERα is required for GEN effect in females and ERβ, as well as PPARγ, in males (34, 35). Even if the sensitivity of the hypothalamus to GEN is well-acknowledged (36, 37), very little is known on neuronal circuits controlling energetic metabolism.

*In vitro*, GEN induces adipocytes' apoptosis, decreases lipid accumulation, and increases lipolysis. Moreover, GEN decreases leptin synthesis (38) and inhibits its secretion (39). *In vivo*, GEN effect depends on sex (40, 41) and on the administered dose (42). In females, an anti-obesogenic effect of GEN is reported for many obese mouse models (43, 44), in juvenile and adult ovariectomized (45, 46) and intact mice (34). This effect is dose dependent (42): GEN inhibits adipogenesis at low concentrations and enhances it at high concentrations (47, 48). GEN effect on fat pad weight is opposite in males, with an obesogenic effect at low doses (34, 35, 49) and an antiobesogenic effect at high doses (34). The effect of GEN during perinatal development may be very different: many studies report an obesogenic effect (50, 51), although only in females (52), while others report an anti-obesogenic effect in males (21, 53). GEN effect during development may be due to epigenetic modifications in the offsprings (54) or to an alteration of the development of estrogen sensitive circuits regulating energetic metabolism, as for other MDCs (11). In fact, GEN is able to affect neural circuits controlling animal welfare and fertility (36, 37), although little is known about its effects on neuronal circuits controlling energetic metabolism. A previous study (21) addressed the effect of soy phytoestrogens, daidzein, and genistein, on the hypothalamus of male mice, reporting that high phytoestrogens levels throughout embryonal and postnatal life decrease AgRP and increase MCH, orexin A and TRH mRNA levels, but it has no effect on NPY, POMC, and CART expression [(21), Figure 1]. While, our ongoing study in male and female mice demonstrates that early postnatal exposure to GEN, in a dose comparable to exposure level in babies fed with soy-based formula, determines an obesogenic phenotype in adult females and a long-term sex specific effects on hypothalamic kiss, POMC and Orexin systems (55). Early post-natal administration of GEN is also influencing the differentiation of other neural circuits in mice not directly related to the control of metabolism (i.e., nitrergic, vasopressinergic, and dopaminergic circuits, [(36), Ponti et al. submitted].

GEN effect on humans is not clear (56). GEN metabolism and bioavailability depends on gut microbioma (57) and GEN exposure may be highly affected by vegan/vegetarian diets (58). The use of soy-based meal replacement formula was effective in lowering body weight and fat mass and reducing LDL cholesterol in obese individuals and together with physical exercise has a beneficial effect on leptin levels in postmenopausal women (59). In contrast, healthy, normal-weight postmenopausal women did not show improvement in metabolic parameters when given high-dose isoflavones (60).

The complexity of the data on the animal and epidemiological studies on the regulation of energetic metabolism, as well as on other neuronal circuits indicate that GEN is a powerful natural compound which may have at the same time highly beneficial or detrimental effects (37) which are worth to be investigated in more detail. Moreover, the contradictory experimental data underline the importance of considering the timing of exposure, the dose/concentration, the sex, and the species-specificity when establishing safety recommendations for dietary GEN intake, especially if in early-life.

### Bisphenol A

Since 1930s, Bisphenol A (BPA) has been involved in the manufacturing of many consumer products [e.g., plastics, PVC, food packaging, thermal papers, (61)]. Thanks to its structure, BPA interacts with a variety of hormone receptors (22): ERα, ERβ, GPR30, and estrogen-related receptor γ [ERRγ, (22)]. Moreover, BPA could also interact with androgen receptor (AR), peroxisome proliferator-activated receptor γ (PPARγ), glucocorticoid receptor (GR), and thyroid hormone receptors [THs, (22)]. These findings strongly suggest that BPA is a multi-target compound that can act on a wide range of hormone-sensitive elements. In fact, BPA has been described also as MDC and the evidences of its role in the alterations of the metabolic axis are increasing (11).

BPA potential obesogenic effects are related to alteration of peripheral parameters, such as weight gain, modifications of leptin or insulin plasma levels, or alterations in the adipose tissue [for a recent review see (62)]. Few studies investigated BPA effects on hypothalamic systems controlling food intake and energy homeostasis, and they are mainly focused on the perinatal exposure (18). BPA exposure of mice from gestational day 0 to Post Natal Day (PND) 21 through diet (1 or 20 μg BPA/kg diet) in combination with HFD had a sexually dimorphic effect on hypothalamic circuits: in males, it impairs glucose tolerance, reduces POMC fiber innervation in the PVN and, in combination with HFD, increases NPY and AgRP expression in the ARC. In females, BPA induces a weight gain, increases food intake, adiposity, and leptin blood levels, while in combination with HFD reduces POMC mRNA expression in the ARC (18). Taken together these data support the idea that BPA acts as a MDC in a sexually dimorphic way [(22), Figure 1].

Gestational BPA exposure (5 mg/L BPA through drinking water) of Sprague-Dawley rat dams increases the proliferation and differentiation of cultured primary hypothalamic neural progenitors (NPCs), as well as the expression of AgRP, while the expression of POMC is reduced (23). BPA is also acting on the kiss system in both rats (63) and mice (64), inducing sexually dimorphic alterations in the cell number of ARC and preoptic populations. Moreover, perinatal treatment with BPA decreases the percentage of kisspeptin-ir fibers in PVN during the postnatal development in female mice (65).

While studies on BPA effects are slowly increasing, only a few studies focus on BPA-analogs: postnatal exposure from PND21 for 10 weeks with 25-50-100 μg/kg BW/day of bisphenol S (BPS) in drinking water affects orexigenic hypothalamic systems resulting in a dose-dependent increase of AgRP mRNA level but not in NPY one or in anorexigenic neuropeptides [POMC, CART; (24)].

Considering the complex relationships between the different circuits involved in the control of food intake and energy homeostasis, further studies are needed to clarify all the effects related to the exposure to BPA and to its less described analogs. In fact, after recognizing the EDC's properties of BPA (66), the search for an appropriate substitute became a fundamental problem to solve. At present, more than 15 BP analogs have been synthesized (67, 68) but none is a real solution. The safety of two of the most used BPA substitutes, BPS and bisphenol F, still remain unclear: *in vitro* and *in vivo* studies, suggests that they share with BPA not only the endocrine-disrupting properties but also the metabolic disrupting ones (69–71).

Both GEN and BPA share a common xenoestrogenic activity, therefore it is possible that they may exert their action altering the estrogens' action on metabolism regulation. In fact, in mammals estradiol (E_2_) has an important role on the regulation of food intake and metabolism with an appetite-suppressing effect (12, 13). In female rodents, ovariectomy (OVX) induces an increased body weight and hyper-adiposity, E_2_ treatment can robustly inhibit food intake (72, 73). Similarly, in our species, women report a decrease in appetite during the periovulatory stage of ovarian cycle, when E_2_ reach a maximal peak (12, 74), while, the development of obesity, type II diabetes and metabolic syndrome in menopause has been correlated with the low E_2_ level (75, 76). These metabolic diseases are partially reverted by E_2_ replacement therapy (77, 78).

E_2_ action is mediated by ERs, in particular, the intracellular ERα, may affect different aspects of regulation of food intake and energy metabolism. This is confirmed by the observation that in rodents deletion of ERα gene cause obesity (79) and the blockage of the appetite-suppressing effect of E_2_ treatment (73). In humans, the polymorphisms in the estrogen receptor alpha gene have been associated with body fat distribution (80). The suppression of ERα expression in VMH alters the anorexigenic effect of E_2_ treatment, leading to obesity, hyperphagia, and reduced energy expenditure in female mice and rats [(81), Figure 1].

Moreover, in ARC and VMH, many neurons co-express ERα and the isoform b of leptin receptor (LepRb) (82). Leptin levels are correlated with E_2_ fluctuation: a decrease of E_2_ reduces leptin secretion, which can be restored by E_2_ treatment (83). Furthermore, both gonadal hormones (84, 85) and leptin (86) modulate Kisspeptin (kiss) anorexigenic neurons. In fact, kiss peptide, co-localizes with ERα (87) and LepRb (88) in ARC. Reciprocal connections link kiss cells, NPY and POMC neurons (89): Kiss excites POMC system directly through the kiss receptor (GPR54) expressed by POMC neurons (90) and inhibits NPY neurons indirectly by enhancing GABA-mediated inhibitory synaptic tone (91). Therefore, hypothalamic kiss system may be a good target for E_2_ in the regulation of food intake and energy metabolism along with the well-known control of reproduction.

Few studies analyzed sexual dimorphism on feeding circuits. The World Health Organization (WHO) reported that the obesity prevalently affects women, and it reaches at twice the rates of men in some regions of the world (92). E_2_ has an important anorexigenic role also in males: the deletion of ERα in mice, (79, 93), as well as the mutation of ERα in men, causes obesity (94, 95) (Figure 1). Moreover, E_2_ treatment in males reduces body weight (4, 96). Sexual dimorphism is reported also for NPY and POMC systems (97, 98) and for their receptors (99–101).

These data support the hypothesis that the metabolic disrupting properties of GEN and BPA as well as of other xenoestrogens are based on their ability in interfering with the estrogenic regulation of metabolism and food intake [reviewed in (11)].

### Tributyltin

Organotin chemicals are compounds containing at least one bond between tin and carbon. The most studied is Tributyltin chloride (TBT), one of the most dangerous and toxic EDC presents in the environment acting as MDC at both peripheral (102) and central level [for recent reviews see (103–106)]. Due to its primarily use in antifouling paint for boats (94), TBT exerted toxicological effects on marine organisms. As a result, fish and fishery products are the main source of human exposure.

Unlike GEN and BPA, Tributyltin chloride (TBT) is an androgen agonist (it binds ARs), while it has no affinity for ERα (107). More recently, TBT has been identified as agonist ligand for RXR and PPARγ (108) and as a promoter of adipogenesis, favoring obesity (109). In fact, PPARγ and RXRγ are strongly express within hypothalamus (110) by nuclei interesting in metabolic and food intake control (as VMH, LH, PVN). Moreover, blocking with pharmacological antagonists or with shRNA the central endogenous activation of PPARγ led to negative energy balance, restored leptin-sensitivity in high-fat diet (HFD)-fed rats (111).

Acute exposure to TBT induced a significant increase of cell expressing c-fos in the ARC nucleus in adult mice (25), thus suggesting a direct action of TBT at the hypothalamic level. A few other studies confirmed this observation. In fact, a chronical exposure to TBT induced a diminution of NPY expression in adult male but not in female mice, a decrease of circulating leptin level, and a decrease of Y1 receptor transgene expression in both sexes (19). Also the POMC immunoreactive system was influenced (26) with a significant decrease of POMC-positive structures in female mice only (Figure 1).

In rats, TBT exposure increased significantly NPY expression in the female together with an increase of food intake, while male presented a decrease of AgRP and CART and appetite (27). Another interesting study in rats investigated whether TBT dependent metabolic disorders were correlated with abnormal hypothalamus-pituitary-gonadal (HPG) axis function, as well as kisspeptin action: after a chronic treatment with TBT, female showed metabolic dysfunctions and HPG axis abnormalities, providing evidence that TBT leads to toxic effects direct on the HPG axis and/or indirectly by abnormal metabolic regulation of the HPG axis (112). TBT has an action also on the hypothalamic-pituitary-adrenal (HPA) axis function (113): a recent study showed that, in female rats, TBT disrupts the morphophysiology of the HPA, leading to an increase in CRH mRNA expression, a decrease in ACTH release and an increase in corticosterone levels (114). Moreover, many studies *in vivo* and *in vitro* have shown TBT effects also on the thyroid morphophysiology and the homeostasis of hypothalamus-pituitary-thyroid axis. TBT may act altering T3 and T4 level (115, 116), down-regulating of thyroid peroxidase, and up-regulating of the thyroid-stimulating hormone receptor (117). TBT given to pregnant mice induces hypothyroidism in the progeny, and induces a dose-dependent increase of T3-independent TRH transcription levels in the hypothalamus of dams (118).

Experimental and epidemiological evidence suggest that the gut microbiota is responsible for significant immunologic, neuronal and endocrine changes that lead to obesity (119), and, recently, it was demonstrated that TBT affect the microbiota system in treated mice, inducing dyslipidemia (120).

In conclusion, TBT has strong effects on both the periphery, with its effects on the mechanisms promoting adipogenesis (121, 122) and the brain by altering the hypothalamic neuroendocrine centers regulating food intake and metabolism (19, 26, 105, 123). All data collected up to now strongly suggest that TBT is a potent MDC.

## Conclusion

In recent years, obesity and metabolic syndromes are increased; even if it is necessary to consider the possible genetic predispositions, and the excessive food intake without appropriate physical exercise, probably the causes should be sought also in numerous natural or synthetic substances that pervade our environment, known as MDCs.

While the possible role as metabolic disruptors of these substances, in particular BPA and TBT, is widely recognized both at hypothalamic and peripheral level, the GEN effect remains controversial on a peripheral level and still unclear, on the hypothalamic neuroendocrine circuits involved in food intake. Therefore, more studies are needed to clarify the interference of these compounds on the complex neural circuit that controls food intake and metabolism.

## Author Contributions

All the authors searched the bibliography. MM wrote a first draft, all the other authors checked for specific part of the manuscript. SG and GCP coordinated the final manuscript.

### Conflict of Interest Statement

The authors declare that the research was conducted in the absence of any commercial or financial relationships that could be construed as a potential conflict of interest.
